# Evaluation of γ-Irradiation Effects on EPDM/SBS Blends for Durability and Recycling Potential

**DOI:** 10.3390/polym17101314

**Published:** 2025-05-12

**Authors:** Traian Zaharescu, Marius Bumbac, Cristina Mihaela Nicolescu, Maria Daniela Stelescu, Tunde Borbath, Istvan Borbath

**Affiliations:** 1 Radiochemistry Center, National Institute for Research and Development in Electrical Engineering ICPE-CA, RO 030138 Bucharest, Romania; traian.zaharescu@icpe-ca.ro; 2 SC Roseal SA, RO 535600 Odorheiu Secuiesc, Romania; borbath.tunde@gmail.com (T.B.); borbathistvan@roseal.eu (I.B.); 3 Faculty of Science and Arts, Valahia University of Targoviste, RO 130004 Targoviste, Romania; 4 Institute of Multidisciplinary Research for Science and Technology, Valahia University of Targoviste, RO 130004 Targoviste, Romania; 5 Leather and Footwear Division, National Research and Development Institute for Textiles and Leather, RO 031215 Bucharest, Romania; dmstelescu@yahoo.com

**Keywords:** ethylene-propylene-diene monomer, styrene-butadiene-styrene, blends, γ-irradiation, degradation, macromolecular interaction

## Abstract

This study investigates the stability behavior of blends composed of ethylene-propylene-diene monomer (EPDM) and styrene-butadiene-styrene (SBS), focusing on the effects of γ-irradiation on these materials. FTIR, CL, and DSC analysis indicate that blends with more than 50% SBS demonstrate remarkable resistance to significant radiation doses. This study highlights that at increased γ-irradiation doses, specifically 100 and 150 kGy, structural changes in the polystyrene aromatic rings are detected, providing insights into the modifications induced by radiation exposure. Among the tested formulations, the blend containing 75% SBS demonstrated the best performance against γ-irradiation, showcasing superior mechanical and structural resistance to radiation-induced degradation. The results indicate that γ-irradiation leads to managed degradation within the SBS/EPDM mixtures: while EPDM experiences increased crosslinking, SBS proves resilient against crosslinking, thus bolstering the stability of EPDM under irradiation scenarios. Additionally, thermal analysis underlines the beneficial role of SBS by showing enhanced thermal stability in SBS-rich samples (SBS content higher than 50%) experiencing reduced thermal degradation through repeated heating cycles. This outcome suggests that the inclusion of SBS effectively reduces crosslinking and chain scission impacts, thereby enhancing consistency in thermal properties over multiple cycles.

## 1. Introduction

The great interest paid to the extended durability of products is addressed to the polymers under various consistencies: mono-components, blends, and nanocomposites of any formulation including macromolecules. The blended compositions win several foreseen properties, whose amplitudes are proportional to the component content [1]. The purposes of polymer blends are correlated with the degrees of behavior modeling regarding the extension of material lifespan. The interdependence of the structural and morphological features and the substrate evolution, the intensity and duration of stress, and the control of the environmental conditions are the essential aging factors that decide the material’s durability [2]. The assistance of radiation exposure makes the initiation of structural transformations induced in the neat polymers possible [3].

The ethylene propylene diene monomer rubber (EPDM) is a generous engineering material that inspires the creation of various molecular configurations by radiation processing. The radiolysis scissions randomly occur. However, the energetic requirements define the double bonds as the weakest interatomic connection due to the low energy of the π bond associated with the highly substituted carbon atoms belonging to the propylene moieties [4]. The ionizing radiation exposure used, applied to improve the functional properties of EPDM, reveals the intrinsic changes caused by the energy transfer onto the target macromolecules [5]. Under certain conditions, the crosslinking of the polymer may be noticed [6]. Otherwise, the macromolecule exposed to a γ ray or electron beam shortens the life span of the material [7]. The functional characteristics and application scope are determined by the blend compositions that include EPDM [8]. The degradation mechanism is initiated by the scission of vulnerable sites existing on the macromolecule, like the bonds and crosslinking points [9].

The radiochemical yields of two polymers are used as a measure to compare their radiation stability when they are combined [10]. In such blends, one polymer acts as the primary source of radicals, while the other predominantly accepts various reactive intermediates, including its own and foreign radicals. The evaluation of the radiation stability of EPDM [11] and SBS [12] suggests that a significant proportion of reactive molecular fragments originates from the ethylene-propylene elastomer. Consequently, EPDM/SBS blends can be vulcanized similarly to PP/SBS systems [13]. The recent overview concerning the radiation effects on polymer blends [14] presents the mechanisms by which free radicals are consumed. Macro radicals and small intermediates react randomly, and their recombination results in stable final structures. The grafting capacity of SBS [15] demonstrates that the most reactive positions available for coupling are located at the α-positions relative to the benzene rings. This indicates that the π-electron delocalization within the benzene rings influences the electron distribution in their vicinity along the macromolecular chains. Meanwhile, the saturated moieties within the ethylene-propylene rubber structure are preferentially attacked at the tertiary carbon atoms. Additionally, the unsaturation present in the butadiene units is similarly vulnerable [16], facilitating the branching of SBS backbones via the grafting of macro-radicals. This conclusion is supported by ESR measurements conducted on various polymers [17], which validate the susceptibility of SBS to molecular chain scission [18].

This study investigates the effects of γ-irradiation on EPDM/SBS blends, with a particular focus on the role of trimethylolpropane triacrylate (TMPTA) as both a crosslinking and compatibilizing agent. By leveraging the environmental resistance of SBS and the free radical generation capacity of EPDM, this research explores an effective strategy for enhancing the stability and processability of these materials. Through complementary investigations, we evaluate the contributions of blended components and illustrate the evolution of γ-irradiated systems, emphasizing the molecular changes that influence material properties. TMPTA has a dual functionality by promoting crosslinking while also enhancing interfacial adhesion between EPDM and SBS. The resulting materials exhibit resilience in γ-irradiated environments, making these radiation-processed formulations promising candidates for high-performance applications, such as sealants, components for oncology devices, nuclear power plants, and other radiation-intensive environments. Furthermore, this approach is particularly relevant for recycling and reprocessing applications, where the adjustment of mechanical and thermal properties is essential after prolonged service life. Our findings demonstrate the potential for tailoring material properties through γ-irradiation, thus enhancing both the performance and recyclability of EPDM/SBS blends. Gamma radiation was chosen as the energy source due to its ability to induce crosslinking in polymer systems with minimal degradation. This is crucial for promoting sustainable polymer disposal and conversion at the end of their lifecycle, thus reducing environmental impact. The study examined SBS/EPDM blend ratios (100/0, 75/25, 50/50, 25/75, 0/100) subjected to γ irradiation at doses of 50, 100, and 150 kGy. The findings provide valuable insights into composition-dependent crosslinking, with the resulting materials demonstrating improved stability and performance. These results lay the foundation for translating experimental data from laboratory research to industrial applications under real-world γ dose exposure scenarios.

## 2. Materials and Methods

### 2.1. Materials

The materials used in the presented study were used as received. Ethylene propylene diene terpolymer (EPDM) Nordel 4760 was purchased from Dow Chemical Company, Dupont Elastomer, Wilmington, DE, USA. This polymer contains 70 wt % ethylene and 4.9 wt % 5-ethylene-2-norbornene (ENB) having a Mooney viscosity of 70 ML_1+4_ at 120 °C, density of 0.88 g/cm^3^ and crystallinity degree of 10%. SBS—Globalprene TM 3545 was provided by LCY Group, Taiwan, China as a linear styrene-butadiene block copolymer, with 43–47% styrene content, 0.94 g/cm^3^, ash content 1%, fluidity index at 190 °C/5 kg of 40–70 g/10 min, hardness 84–88 °Sh. As the crosslinking coagent, trimethylolpropane trimethacrylate (TMPT) ALCANPOUDRE TMPTMA-70, Safic-Alcan UK Limited, Warrington, UK, was used. The crosslinking agent ALCANPOUDRE TMPTMA-70 is a mixture of 70% TMPT, the active ingredient, with 30% precipitated silica.

### 2.2. Preparation and Processing of Samples

The samples were prepared using the melt blending technique using an electrically heated laboratory roller mill machine, model ZG-160YRDB (from Xiamen Ollital Technology Co Ltd., Xiamen, China). The working parameters were: friction ratio 1:1.2, temperature 95–115 °C, and blending time 10 min for each formulation. The sample compositions are listed in Table S1 (Supplementary Material—Section S1). The sequence of operations started with the addition of SBS into the roller mill and melted for 1–2 min; then EPDM was added and melt-blended for 2 min in the mill. When a homogeneous mixture was obtained, TMPTMA was added for 1–2 min. Finally, the composition was homogenized for 5 min and removed from the roll-in sheets of about 2 mm thickness.

TMPTA acts as a crosslinking agent, enhancing the formation of crosslinked networks within the polymer blend under irradiation, and also acts as a compatibilizer by improving the interfacial adhesion between EPDM and SBS. EPDM and SBS are inherently immiscible due to their different chemical structures (EPDM being a non-polar elastomer and SBS being a block copolymer with polar and non-polar segments). TMPTA can reduce phase separation and enhance the homogeneity of the blend, leading to improved mechanical and thermal properties.

The resulting mixtures were used to obtain plates (300 mm × 300 mm × 2 mm) by compression molding, using the laboratory electrical press (Fortune Presses model no. TP 600 manufactured by Fontijne Grotnes, Vlaardingen, The Netherlands) and frame-type molds. The processing parameters were carefully selected: sample preheating for 5 min, at the temperature of 160 °C and the contact pressure, modeling at the temperature of 160 °C and the pressure of 5 MPa for 4 min followed by the cooling down stage to 45 °C for 10 min and the pressure of 5 MPa. The obtained plates were stabilized for 16 ore at room temperature before the preparation of dumbbells. The test samples were punched from the obtained plates using an automatic punching machine.

The γ-exposure was accomplished in an irradiation machine equipped with a ^60^Co source at a dose rate of 0.5 kGy h^−1^ in air at room temperature. The circular system assured the equally exposed process. The measurements were performed soon after the end of irradiation, preventing the decay of short-life radicals.

### 2.3. Chemiluminescence Study

The thermal stability of processed samples was evaluated by chemiluminescence, whose high accuracy allows the characterization of the oxidation degree under isothermal (160 °C, 170 °C, and 180 °C) and non-isothermal (10 °C min^−1^) conditions. The proportionality between the oxidation degree expressed by the number of emitted quanta and time or temperature, respectively, affords the discussion on the evolution of oxidation states according to the deexcitation mode [19].

The activation energies required for the oxidation of the studied samples were calculated by the Arrhenius procedure presented in Section S2 of the Supplementary Material. 

### 2.4. Analysis of the Gel Fraction (Equilibrium Solvent Swelling Measurements)

The determinations were made at room temperature using toluene as solvent. These were weighed (***m_i_***) and then immersed in toluene for 72 h. After immersion, the excess solvent was wiped off, and the samples were immediately reweighed (***m_s_***). The solvent was removed from the samples by drying in the open air (3 days) and then in an oven with forced air circulation at 120 °C for 4 h, and finally, the samples were weighed again (***m_d_***). The gel content is calculated as the percentage mass fraction of the insoluble part of the rubber, using Equation (1) [20]:

(1) $$\mathrm{Gel}\;\mathrm{fraction}\;\left(\%\right)=\frac{m_{d}}{m_{i}}\times 100$$

This procedure was applied for four specimens whose averages were taken into consideration for the evaluation of gel fraction.

The crosslinking density was calculated by using the Flory–Rehner Equation (2) [21,22]

(2) $$\nu_{cross}\left(\frac{mol}{g}\right)=-\frac{ln\left(1-V_{r}\right)+V_{r}+\chi_{12}V_{r}^{2}}{V_{s}\left(\sqrt[{3}]{V_{r}}-V_{r}/2\right)}$$

where ν_cross_ is the crosslinking density, χ_12_ is the polymer/solvent interaction parameter, *V_s_* is the molar volume of the solvent used (106.13 cm^3^ mol^−1^ for toluene) and *V_r_* is the volume fraction of rubber in swollen mass calculated with the equation presented in Section S3 of the Supplementary Material [23,24].

By applying the Charlesby–Pinner representation (Equation (3)), the dissolution behavior of the γ-irradiated blends is evaluated using the mathematical relation (3):

(3) $$s+\sqrt{s}=\frac{p}{q}+\frac{0.96\times 10^{7}}{q\cdot {\bar{M}}_{n}}\frac{1}{D}$$

where *s* is the sol fraction of irradiated samples, *p* is degradation density, *q* is crosslinking density for each absorbed 100 eV, ${\bar{M}}_{n}$ is initial molecular weight and *D* is radiation dose (kGy).

### 2.5. Physical and Mechanical Testing

The samples destined for the evaluation of physical properties were conditioned according to ISO 23529 [25]. Specific gravity (ISO 2781) was obtained by means of a VF4601 density kit mounted on Secura225D-1CEU balance (Sartorius, Göttingen, Germany).

The mechanical testing was achieved using the equipment ZMR 250 (VEB THURINGER, Suhl, Germany), applying the testing standard ISO 37 [26]. The evaluation of ° Shore A hardness was based on ISO 48-4 [27] with testing unit equipment from Zorn Instruments GmbH, Stendal, Germany.

### 2.6. FTIR Analysis

The spectral data were recorded on Vertex 80 infrared spectrometer (Bruker, Karlsruhe, Germany) provided with an ATR investigation system. The scanning range was 4000 cm^−1^ to 400 cm^−1^ at a spectral resolution of 4 cm^−1^ and 32 scans.

### 2.7. DSC Analysis

The thermal analysis system DSC3-StarE (Mettler Toledo, Greifensee, Switzerland) was used to record DSC thermograms of polymer samples. Tests were run in the temperature range of 30 to 300 °C at a heating rate of 10 °C min^−1^. The samples having a mass of 10 ± 1 mg were weighted on Secura 225D-1CEU (Sartorius, Göttingen, Germany) analytical balance with a precision of 1 × 10^−5^ g placed in standard aluminum pans of 40 µL. An empty 40 µL aluminum pan was used as a reference.

## 3. Results and Discussion

The γ-exposure of studied blends may be considered a proper method for the valuation of deep structural modification when the two main competitive processes that occur are engaged in a simultaneous but competitive processes: recombination and oxidation [28]. The fundamental peculiarity of polymer radiolysis concerns the molecular fragmentation followed by the radical decay routes [4]. The high energy exposure makes it possible to make polymer blends compatible even if the components are immiscible [29] or to test the oxidation resistance under certain special circumstances [30].

### 3.1. Chemiluminescence

The chemiluminescence measurements provide an accurate evaluation of the progress in the oxidation state of polymers by recording the photons emitted by oxidizing radicals. Any modifications occurring in the structure of the polymer substrates are appropriately visualized in the chemiluminescence spectra, due to the correlation between the accumulation of oxidation intermediates (peroxy radicals as precursors of final oxidation products) and the recorded chemiluminescence intensities [31].

#### 3.1.1. Isothermal Chemiluminescence

**Figure 1:**
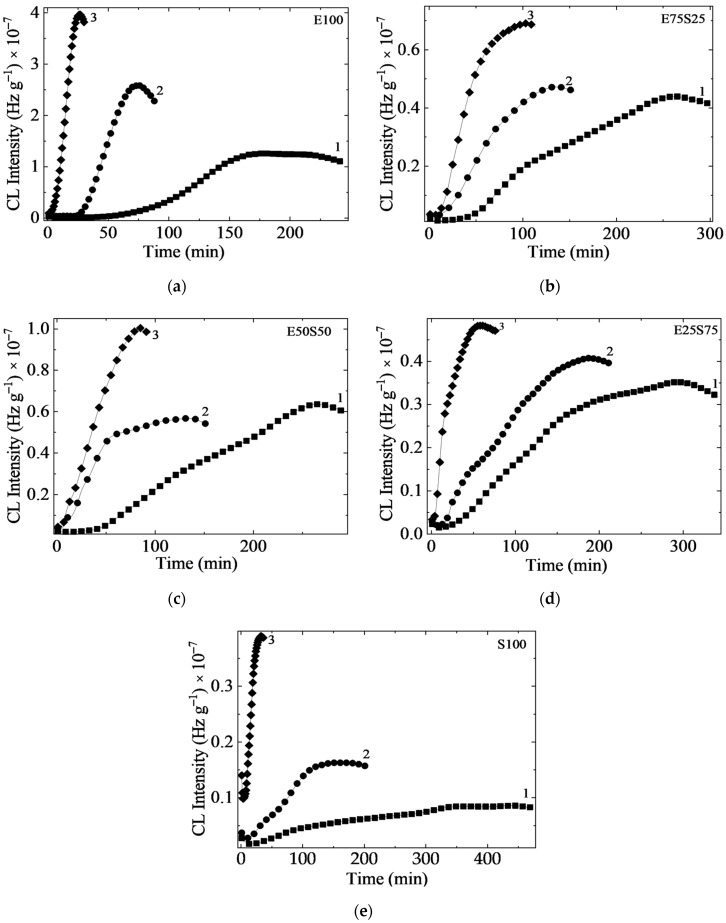
The isothermal chemiluminescence spectra recorded on EPDM/SBS systems: (**a**) E100, (**b**) E75S25, (**c**) E50S50, (**d**) E25S75, and (**e**) S100. Degradation temperatures: (1) 160 °C—square, (2) 170 °C—circle, (3) 180 °C—diamond.

The radiolysis of blended components, EPDM [32] and SBS [33] reveals the main radicals that are involved in the degradation chain of each phase. Though the evolution of the oxidation state of polymer blends is controlled by the diffusion of oxygen into the deeper zones of samples, a certain contribution to the material stability is yielded by the crosslinking of intermediates [34]. The isothermal chemiluminescence measurements (Figure 1) afford the assessment of oxidation strength according to the mechanism of oxidation based on the self-catalytic process [35]. Several oxidation features describe the material resistance against degradation:

- *The oxidation induction time* (Table 1) indicates the effective start of the process when the measurable quanta number in the start of the propagation step sustained by the reactions of free radicals with molecular oxygen or peroxyl entities is recorded. It means that a sufficient amount of oxygen is available and the concentration of radicals may feed the oxidation chain;
- *The oxidation rate* suggested by the curved slope characterizes the decay of oxidized intermediates when the ability of the included phases for the molecular scission allows the accumulation of reactive fragments to feed the aging process. The stability difference between the two polymers depicts the unlikely progress of oxidation when the blends are subjected to an energetic transfer.

Under energetic conditions, radical generation is adjusted, with weaker bonds being randomly broken. However, the presence of benzene nuclei in SBS restricts styrene fragmentation [36]. Comparing the oxidation progress of EPDM and SBS reveals that a higher proportion of EPDM worsens oxidation resistance, as supported by radiation stability assays, indicating that EPDM/SBS blends with 80% EPDM are the most susceptible to degradation when exposed to 15 kGy electron beam irradiation [37]. Thermal stability analysis of EPDM/SBS blends (Figure 1) further highlights compositional differences arising from simultaneous crosslinking and oxidation processes.

The strength of the structure achieved by the studied blends is demonstrated by the activation energy (Ea) values calculated using the Arrhenius equation [38]. The calculated Ea values are presented in Table 1. These values are similar to other reported results [39]. The decrease in the Ea values as the SBS content increases indicates an effective method for recycling polymer wastes that contain polyolefins, such as EPDM. The substantial decrease in Ea values for the oxidation process of the tested samples can be specifically attributed to the butadiene segments present in the SBS structure [40].

**Figure 2:**
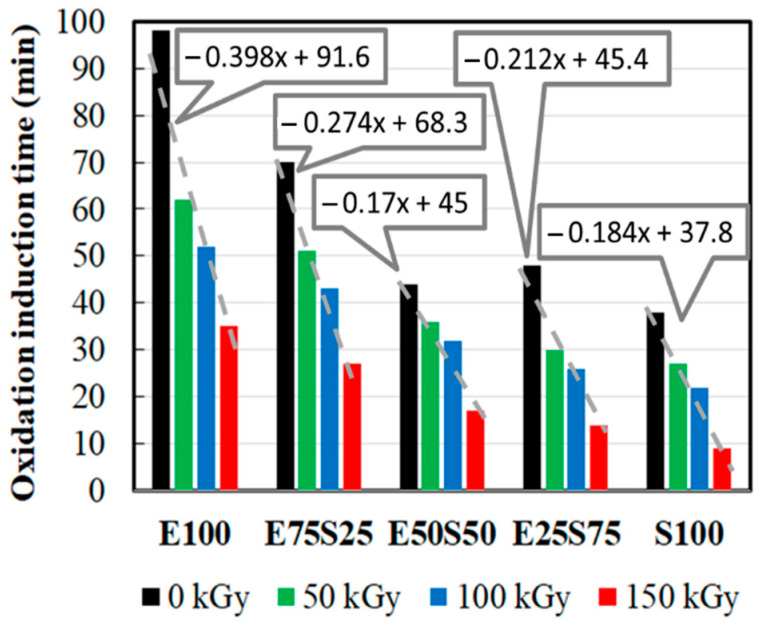
The oxidation induction times measured by isothermal chemiluminescence accomplished at 160 °C at all applied γ-doses.

Figure 2 shows the decrease in the thermal resistance of irradiated blends when they are exposed to the energetic transfer from incidental rays onto molecular backbones. As may be anticipated for the tested mixtures, the induced degradation is more pronounced for samples where the SBS consistency is higher. This effect may be associated with the higher crystallinity degree of EPDM, which mitigates the faster dissipation of energy [41]. Thus, the presence of SBS allows faster oxidation when the materials are destined for the conversion of waste state into useful items. The propagation of oxidation is conditioned by the blending ratio, which reveals the proportional contribution of each type of radiolysis fragment either on the initiation of oxidation chains or on the extension of the degraded fraction.

Chemiluminescence measurements conducted during isothermal treatment at 160 °C revealed a consistent linear decrease in the oxidation induction time across all samples with increasing radiation doses. This feature suggests that the irradiation makes the polymers more susceptible to oxidative degradation, regardless of the blend composition.

Analyzing the slope of the linear trend lines showed that the impact of radiation dose on oxidation tendency is most pronounced in EPDM, as evidenced by its steepest negative slope. In contrast, the E50S50, E25S75, and S100 samples exhibited similar negative slope values. Notably, increasing the SBS content beyond 50% in the polymer blend confers a specific behavior similar to that of S100, indicating that the changes in crystallinity and the number and type of scission sites in the polymer had little effect on the radical propagation mechanism during irradiation.

**Figure 3:**
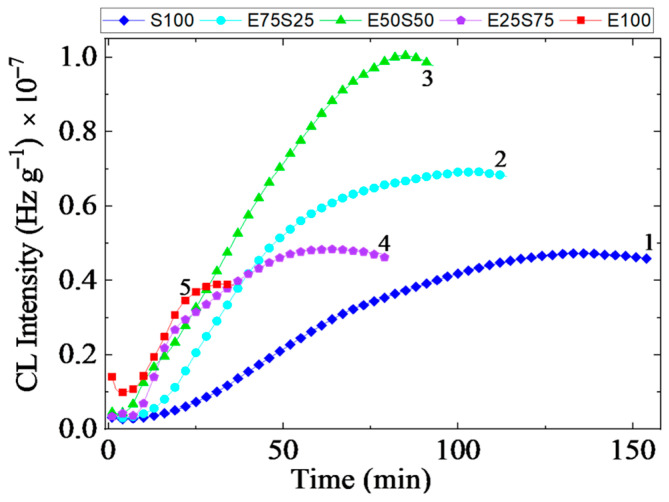
The isothermal CL spectra of γ-irradiated samples (Dose: 150 kGy. Testing temperature: 160 °C): 1—S100, 2—E75S25, 3—E50S50, 4—E25S75, 5—E100.

During irradiation, a certain number of new bridges are formed [42], but γ-exposure also causes their partial breakage at a defined rate [43], as shown by the curve ascensions in Figure 3. Figure 3 illustrates the involvement of radicals, highlighting the participation of intermediates in either increasing the CL intensity—indicating reactions within the same phase—or interphase spreading as the SBS loading increases. The influence of irradiation dose on the components is reflected in the variation in structural moieties [44,45], which affect the recombination rate by altering oxygen transport through the material [35].

As illustrated in Figure 3, the CL intensity exhibits a turning point above 50% EPDM content. Samples with EPDM concentrations exceeding this threshold show notably lower CL intensities after γ-irradiation at 150 kGy, suggesting a potential shift toward thermoset behavior. This is attributed to the increased crosslinking density imparted by EPDM, which restricts chain mobility and reduces oxidative degradation, resulting in diminished CL signals. In contrast, thermoplastics typically display higher CL intensities due to their greater chain mobility and susceptibility to oxidative degradation, as reflected by the elevated CL signals observed in samples with lower EPDM content, indicative of chain scission and volatile product formation.

#### 3.1.2. Nonisothermal Chemiluminescence

**Figure 4:**
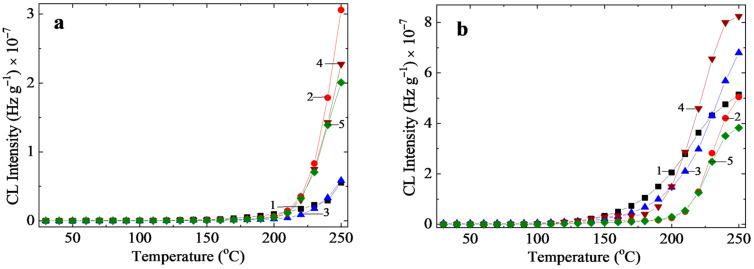

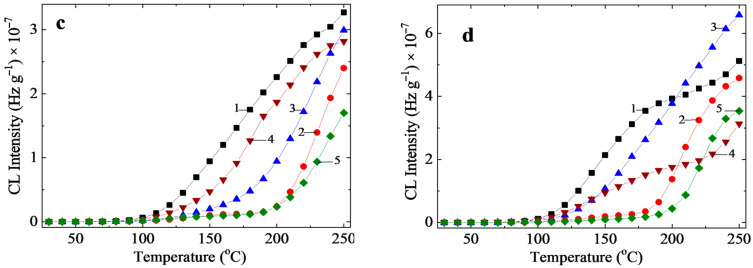
The nonisothermal CL spectra recorded at the heating rate of 10 °C min^−1^ for samples irradiated at: (**a**) 0 kGy, (**b**) 50 kGy, (**c**) 100 kGy, and (**d**) 150 kGy, where (1)E100—black square; (2) E75S25—red circle; (3) E50S50—blue triangle; (4) E25S75—purple triangle; (5) S100—green diamond.

The general survey on the investigation of stability by nonisothermal chemiluminescence indicates the destructive influence of γ-exposure on molecular integrity (Figure 4). The influence of the early decomposition of EPDM is suggestively illustrated at an exposure dose exceeding 100 kGy by the initial start of its oxidation. A notable feature of the radiation resistance of SBS is its longer onset oxidation temperature (OOT) of 183 °C at 150 kGy, compared to the values obtained for other samples under similar conditions (Table 2). The lowest OOT values found for EPDM indicate the initiation of degradation at an early stage, where EPDM molecules are broken down at the diene units. In contrast, the unexpectedly high OOT values for the blend E75S25 reflect the influence of benzene structures on the significant concentration of radicals from EPDM, while the integrity of the SBS molecules remains unchanged. According to the binding effect of SBS in the polyolefin blends enhanced by γ-irradiation [44], its structure is suitable for reducing the level of oxidation.

As presented in previous reports [46,47], the sensitivity of EPDM to molecular scission surpasses that of SBR fragmentation. The early onset of oxidation in all nonisothermal CL determinations on the ethylene-propylene-diene monomer (Figure 4, Table 2) indicates the initiation of degradation in this component, establishing EPDM as the primary source of free radicals. This initiation reflects the instability of all studied blends. However, samples with a higher content of SBS demonstrate greater stability, with E25S75 being the most representative. Formulations with a higher proportion of EPDM are more suitable for sensitizing blends during γ-irradiation. An increase in the applied dose above 50 kGy results in faster oxidation even at lower temperatures, significantly contributing to the oxidation states.

The non-isothermal chemiluminescence (CL) measurements provide an assessment of the thermal stability of irradiated samples, showing that oxidation begins at lower temperatures as the exposure dose increases. While the E100 and S100 samples exhibit measurable oxidation at higher temperatures, around 150 °C, the samples exposed to a dose of 150 kGy display oxidation intensities at temperatures below 100 °C (Figure 4). These observations about sample stability emphasize the underlying mechanisms through which the studied mixed components contribute to the development of oxidation levels. As shown in Table 2, the onset oxidation temperature increases with the SBS content, highlighting the improved oxidative stability of blends with higher SBS concentrations. However, samples composed solely of EPDM are the most prone to oxidation, even without irradiation, due to their lower onset oxidation temperature. This is particularly critical considering the processing temperature of approximately 180 °C in the extrusion machine, where EPDM-only samples are more likely to undergo oxidative degradation.

### 3.2. Analysis of the Gel Fraction

Swelling experiments, involving the diffusion of toluene into samples with various compositions, were conducted on all sample types. However, all non-irradiated samples, as well as irradiated samples with an SBS content exceeding 50 wt% (regardless of the radiation dose), dissolved completely in toluene, making further analysis impossible. Non-irradiated samples dissolved due to the low level of crosslinking in the material. In the case of irradiated samples with high SBS content, this dissolution occurs because the polybutadiene regions in SBS swell or dissolve in toluene owing to their high compatibility with the solvent. Unless a robust crosslinked network is formed to stabilize the polymer chains, the SBS structure disintegrates, leading to complete dissolution. Gamma irradiation induces crosslinking in polymers by creating covalent bonds between polymer chains. However, the efficiency of crosslinking strongly depends on the polymer composition. For SBS-rich samples (SBS content > 50 wt%), γ-irradiation appears to result in insufficient crosslinking, which leads to their dissolution. This suggests that SBS does not crosslink as effectively as EPDM under γ-irradiation. The softer polybutadiene blocks in SBS may degrade or undergo scission more readily under irradiation, weakening the polymer structure. Consequently, some polymer blends fail to develop a stable crosslinked network and dissolve completely in toluene during swelling experiments.

The gel fraction, crosslinking density, and volume fraction of rubber in the swollen mass increase with the EPDM content, which can be attributed to the radicals generated primarily by the EPDM elastomer. Although the density variation among the samples is not significant (as shown in Table 3), the increase in the degree of crosslinking demonstrates the availability of radicals to form intermolecular bridges, particularly in the presence of the multifunctional monomer TMPTMA. A comparison of the crosslinking density (ν_cross_) values for blends with decreasing SBS content reveals that the monomer functions as a radical scavenger, assisting in recombination rather than the oxidation of radicals. It can be inferred that the breaking of SBS chains at tertiary carbon atoms produces radicals with delocalized electrons in the benzene structure, which are less likely to participate in the crosslinking process.

**Figure 5:**
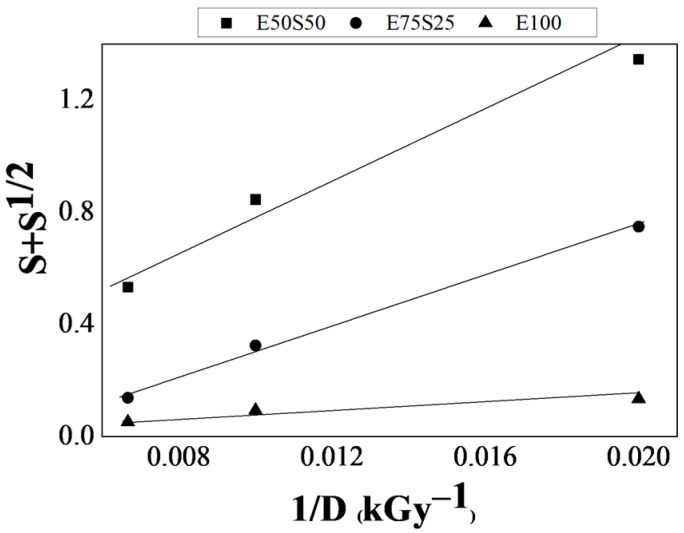
Charesby–Pinner representation for γ-irradiated some EPDM/SBS blends, (square—E50S50, circle—E75S25, triangle—E100).

The effects of irradiation on the EPDM/SBS blends can also be evaluated by analyzing the ratio of *p/q*, where *p* is the radiochemical yield of chain scission, and *q* is the radiochemical yield of crosslinking, as determined using the Charlesby–Pinner equation [48]. The calculated ratios are presented in Table 3. A decrease in the slope values, calculated using Equation (3), indicates a more significant contribution of crosslinking, as shown in Figure 5.

As the proportion of EPDM increases, the ratio (*p/q*) decreases, indicating the involvement of radicals generated from EPDM and a greater resistance to scission of the polymer chain when the blends are irradiated. This behavior can be attributed to the responses of the polymer chains under irradiation: the butadiene chains in SBS are more prone to scission, while the polyethylene and polypropylene chains in EPDM are more inclined to crosslink [49]. Additionally, at 150 kGy, advanced fragmentation is caused by the sustained penetration of oxygen due to its higher diffusion rate, contributing to the degradation of SBS-rich samples. The results presented in Figure 5 align with the CL data in Figure 3, where the rise in CL intensity is associated with an increased degree of polymer crosslinking during the irradiation process. It is important to note that the S100 and E25S75 samples were completely dissolved in toluene, regardless of the irradiation dose, indicating the absence of a measurable solid fraction even at 150 kGy. If irradiated EPDM were present in the sol fraction after dissolution, a measurable sol fraction would be expected at higher doses, particularly at 150 kGy. However, this was not observed, suggesting that the sol fraction does not exclusively consist of irradiated EPDM.

Further analysis of the (*p/q*) ratios (intersection of the graph with the Ox axis) reveals an interesting trend: the (*p/q*) values for E100 and E75 samples are close to each other, while the E50 sample exhibits a significantly higher (*p/q*) value, approximately five times greater. This suggests a potential inflection point in the behavior of the polymer blend around 50% EPDM content. Above this threshold, crosslinking appears to accelerate significantly, as evidenced by the fact that (*p/q*) ratios for E75 and E100 samples are about the same. This trend is consistent with the greater crosslinking propensity of EPDM under irradiation, likely due to the chemical structure of its polyethylene and polypropylene chains. While it cannot be definitively concluded that the sol fraction contains only irradiated EPDM, the data strongly suggest that crosslinking becomes more dominant as the EPDM content increases, particularly beyond 50%. This observation aligns with the reduced (*p/q*) ratios at higher EPDM concentrations and irradiation doses. 

### 3.3. Mechanical Testing

According to the degradation study [50], the SBS component generates sufficient radicals during the initiation stage, which are then involved in radical–radical recombination reactions during the propagation stage. It has been reported that when SBS undergoes scission, it separates into two distinct, immiscible phases: polybutadiene (the rubbery segment) and polystyrene (the glassy segment). These two phases do not mix well due to their differing chemical properties. As more energy (e.g., from irradiation) is applied to the material, bond-breaking increases, reducing the material’s cohesion and structural integrity [51].

The structural modifications in irradiated samples are dependent on the degree of scission, which impacts the material’s elasticity [52].

**Figure 6:**
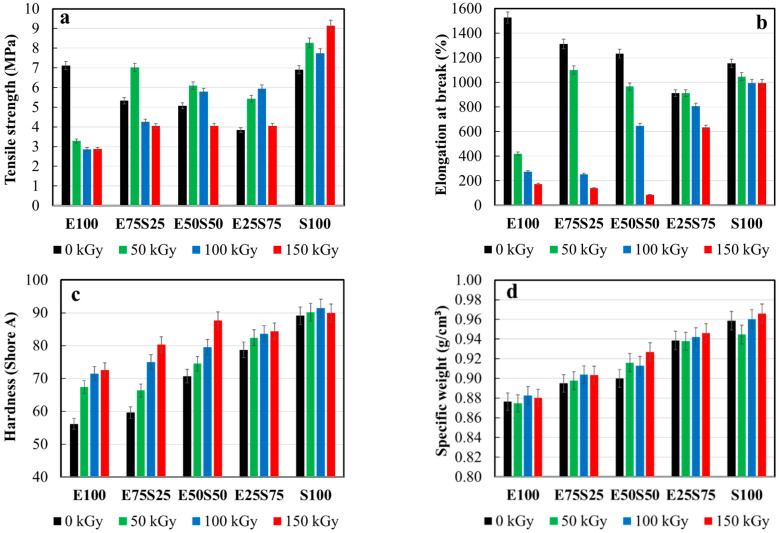
The physico–mechanical testing results: (**a**) tensile strength, (**b**) elongation at break, (**c**) hardness (Shore A), and (**d**) specific weight.

The decrease in EPDM content leads to a reduction in tensile strength for non-irradiated samples. Specifically, E100 and S100 samples exhibit similar tensile strengths, while the tensile strength hierarchy for blended samples is E100 > E75S25 > E50S50 > E25S75 (Figure 6). As the irradiation dose increases, particularly noted in Figure 6, the tensile strength of E100 decreases dramatically. In contrast, γ-irradiation at 50 kGy induces a slight increase in tensile strength for the other samples, but higher doses of γ-irradiation (>100 kGy) result in a further decrease. Notably, at 150 kGy, the tensile strength of these irradiated samples falls below that of the non-irradiated counterparts. This decline is attributed to a higher crosslinking density in the polymer matrix, which negatively affects the degree of crystallization [53,54].

While the material hardness decreases gradually, prolonged irradiation causes a significant reduction in elongation at break and tensile strength. This behavior is primarily attributed to molecular fragmentation. At early irradiation stages, up to 50 kGy, the damaging effects of degradation on morphological features are partially balanced by random recombination, as the gel dose for SBS is only 6 kGy [55].

The addition of a crosslinker helps limit the severe degradation of mechanical properties by increasing the crosslinking density [56]. However, the semi-crystalline structure of EPDM restricts radical mobility [57]. Consequently, exposing the tested blends to higher irradiation doses leads to advanced aging, characterized by diminished mechanical resistance.

The elongation at break decreases as SBS content increases. Mixtures containing more than 75% SBS become noticeably less ductile, as shown in Figure 6b. For blends with more than 50% EPDM, the elongation at break decreases sharply upon γ-irradiation. This behavior suggests that EPDM promotes extensive crosslinking under radiation. In contrast, blends with a higher SBS content (>75%) exhibit greater resistance to radiation-induced crosslinking, retaining more of their original ductility (Figure 6b). At lower radiation doses (50 and 100 kGy), the E50S50 blend maintains elongation at break better than the E25S75 or E100 blends. This suggests that the 50:50 blend achieves an optimal balance between crosslinking and ductility at moderate radiation doses.

Additionally, increasing SBS content improves the hardness and specific weight of the material, indicating that SBS contributes to a denser and stiffer composite structure (Figure 6c—black bars). For mixtures with more than 50% EPDM, irradiation significantly increases hardness due to advanced crosslinking. However, this makes EPDM-dominant blends harder but also more brittle, which limits their applicability in dynamic environments.

The reported data align with the sol-gel assay results, as higher SBS content leads to greater variations in the *p/q* ratio (Table 3), reflecting the formation of a more robust crosslinked network in samples with lower SBS content during irradiation.

Finally, the specific weight remains largely unaffected by radiation dose and is primarily determined by SBS concentration. Higher SBS content correlates with increased density, regardless of irradiation levels.

### 3.4. IR Analysis

**Figure 7:**
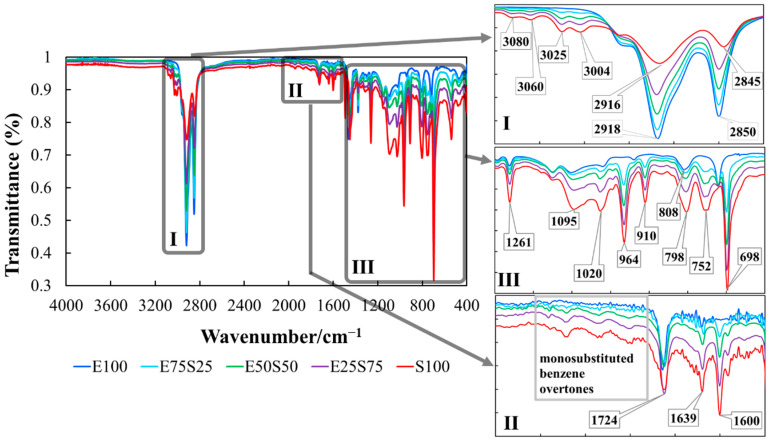
IR spectra of non-irradiated samples with different content of SBS (I—inset zoom in the wavenumber range 3200–2800 cm^−1^; II—inset zoom in the wavenumber range 1800–1500 cm^−1^; III—inset zoom in the wavenumber range 1500–400 cm^−1^.

The trends of modifications in the recorded IR spectra are related to the specific functional groups and structural components of SBS and EPDM from the polymer blends (Figure 7).

The key information extracted from the spectra presented in Figure 7 is presented below:

***Non irradiated samples***

*O-H stretching region (3100–3600 cm^−1^):*

- A weak band is observed in all samples due to the presence of TMPTMA as a crosslinking agent. The band persists even at low TMPTMA concentrations (3%), likely due to trace residual hydroxyl groups or slight hydrolysis.
- Its consistent presence across all spectra suggests it originates from TMPTMA, not blending byproducts in non-irradiated samples.

*C-H stretching vibrations (aromatic and olefin groups):*

- Bands at 3080, 3060, 3025, and 3004 cm^−1^ correspond to C-H stretching in styrene (aromatic) and butadiene (olefin) groups. Intensity increases with SBS content, making these bands markers for SBS proportion.

*Aliphatic C-H stretching:*

- Bands at 2916 and 2845 cm^−1^ represent symmetric and asymmetric stretching of aliphatic C-H bonds. Intensity decreases with increasing SBS content, reflecting reduced ethylene proportion.
- Slight band shifts (2916 to 2918 cm^−1^ and 2845 to 2850 cm^−1^) indicate changes in intermolecular interactions due to SBS replacement by EPDM.

*Mid-IR aromatic and aliphatic components (insets II and III):*

- Band at 1600 cm^−1^: C = C stretching in the benzene ring of SBS styrene; intensity increases with SBS content, serving as a marker for aromatic styrene [58,59].
- Bands at 1465 and 1450 cm^−1^: C-H bending (scissoring) in saturated aliphatic chains; intensity remains consistent as both SBS and EPDM contain aliphatic C-H groups.
- Band at 1377 cm^−1^: C-H bending (rocking) in methyl/methylene groups; absent in S100 due to the lack of EPDM-specific aliphatic environments or band suppression at 100% SBS.
- Bands at 1094, 1024, and 964 cm^−1^ correspond to C = C stretching and bending in butadiene and styrene units, reflecting substitution patterns and C=C bond environments.
- Bands at 798, 752, and 698 cm^−1^ represent C-H out-of-plane bending in substituted benzene rings, confirming the aromatic styrene component in SBS.
- Band at 698 cm^−1^, specific to benzene C-H vibrations, disappears in EPDM-only samples, serving as a marker for SBS aromatic content. Thus, the absence of this band in SBS-free samples could serve as a clear marker for the aromatic content of SBS aromatic content [60,61].

***Structural change in the blend upon irradiation***

**Figure 8:**
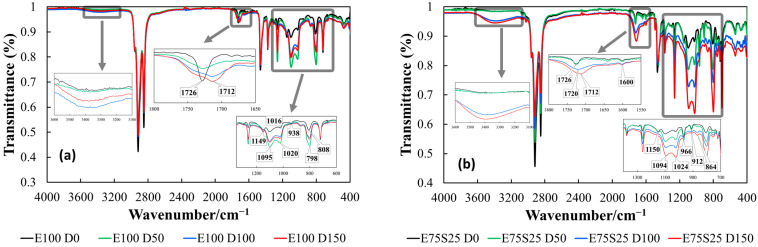

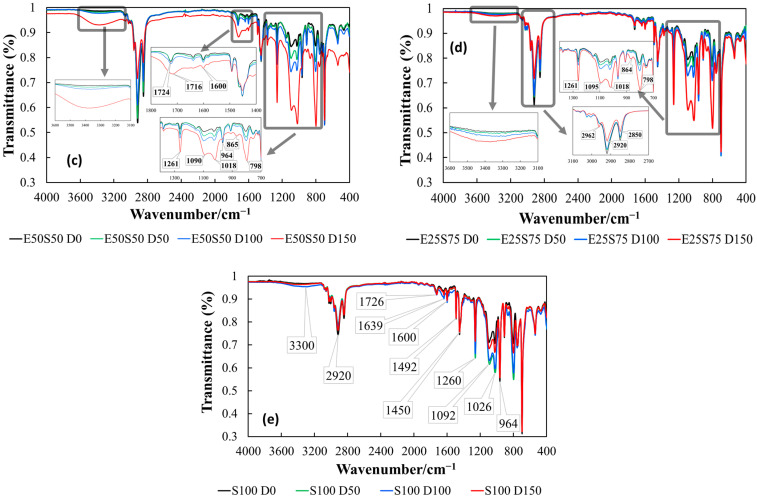
IR spectra of composites of non-irradiated and irradiated samples for: (**a**) E100, (**b**) E75S25, (**c**) E50S50, (**d**) E25S75, and (**e**) S100 (black line—0 kGy, green line—50 kGy, blue line—100 kGy, red line—150 kGy).

Figure 8 presents the IR spectra of the polymer blend samples before and after irradiation, with insets highlighting areas of significant change.

*O−H stretching absorption band (3100–3600 cm^−1^):*

- The intensity of the O−H band increases with radiation dose, varying based on the SBS content in the mixtures [58,59].
- Radiation dose influence.
- Low doses (up to 50 kGy)—minimal changes in O−H intensity.
- High doses (100–150 kGy)—significant increases in O−H intensity, particularly in high-EPDM-content samples, E100 and E75S25 (Figure 8a,b). E50S50 mixture registers a noticeable rise at 150 kGy (Figure 8c).
- High-SBS-content samples, E25S75 and S100 (Figure 8d,e), register minimal changes, indicating higher resistance to radiation-induced oxidation.

*C=O vibrations bands (1700–1750 cm^−1^):*

- Irradiation causes a peak shift toward 1712–1716 cm^−1^.
- Broadening of the absorption band due to the formation of carbonyl groups (e.g., ketones, carboxylic acids).
- Changes are more pronounced in samples with less than 50% SBS, while E25S75 and S100 blends show minimal changes, suggesting greater resistance to forming C=O structures. A higher reactivity of saturated carbon chains in EPDM contributes to C=O generation, while SBS resists oxidation due to the stabilizing effect of its styrene components [60,61].

*C−O bond vibrations (1090 and 1020 cm^−1^):*

- High-EPDM-content samples (E100, E75S25, E50S50) show significant increases in absorbance with rising radiation doses. These bands indicate radiation-induced crosslinking and/or oxidative degradation.

Substituted epoxides or cyclic ethers (1261 cm^−1^) [62]:

- The intensity of this band increases with radiation dose, suggesting the formation of epoxy structures [62].

*Structural degradation in polyolefin structures [61,63]:*

- Band at ~1150 cm^−1^ (prominent in high-EPDM-content samples) decreases in intensity with increasing γ-radiation dose and disappears entirely at doses of 100–150 kGy.
- Band at 938–966 cm^−1^ (saturated polyolefin structures) disappears in samples containing more than 50% EPDM.

These bands, attributed to vibrations characteristic of saturated polyolefin structures, reflect structural degradation caused by chain scission or other changes induced by irradiation.

These spectral changes align with trends observed in Chemiluminescence (CL) testing, analysis of the gel fraction, and mechanical testing results.

**Figure 9:**
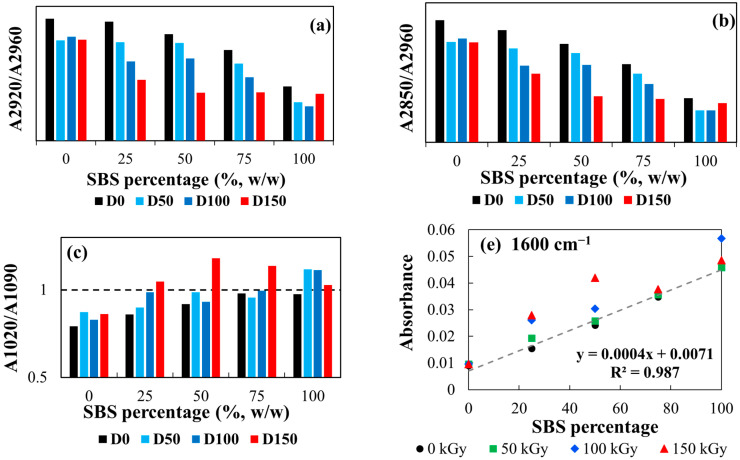
Qualitative analysis of molecular changes reflected by the IR spectra by representing absorbance ratios vs. radiation dose: (**a**) A2920/A2960, (**b**) A2850/A2960, (**c**) A1020/A1090, and (**d**) absorbance measured at 1600 cm^−1^ (A1600) vs. SBS content (black—0 kGy, green—50 kGy, blue—100 kGy, red—150 kGy).

The IR analysis reveals changes in the absorbance ratios at 2850 cm^−1^ (symmetric stretching of –CH_2_ groups), 2920 cm^−1^ (asymmetric stretching of –CH_2_ groups), and 2960 cm^−1^ (asymmetric stretching of C-H bonds in –CH_3_ groups). Among these, the peak at 2960 cm^−1^ remains almost constant across the IR spectra of all samples and is used as a reference point for normalizing the absorbances. Accordingly, the absorbance ratios A2920/A2960 and A2850/A2960 (as shown in Figure 9a,b) were analyzed as a function of radiation dose to evaluate the structural changes induced by γ-irradiation in the material. These ratios serve as sensitive indicators of structural changes in saturated polyolefin chains caused by both the blending composition and γ-irradiation.

For non-irradiated samples, both absorbance ratios decrease almost linearly with increasing SBS content. This behavior suggests that SBS contributes to structural changes, reducing the absorbance at 2920 cm^−1^ and 2850 cm^−1^. This is because SBS has fewer –CH_2_ groups compared to EPDM, which contains a higher proportion of aliphatic chains [61,63]. Furthermore, γ-irradiation induces a decrease in both absorbance ratios, regardless of dose, indicating that irradiation causes structural modifications within the compound. Notably, the absorbance ratios in E100 and S100 samples stabilize after a dose of 50 kGy, suggesting that further crosslinking or degradation of aliphatic saturated chains is minimal beyond this point. In contrast, for the mixtures (E25S75, E50S50, E75S25), the absorbance ratios decrease linearly with increasing radiation dose, pointing to progressive, dose-dependent structural changes.

The stabilization of these ratios in unmixed samples (E100 and S100) beyond 50 kGy may indicate a saturation point for chain scission or crosslinking in the individual polymers. On the other hand, the linear decrease in the absorbance ratios in blended samples (E25S75, E50S50, E75S25) with increasing radiation dose reflects the influence of interactions between SBS and EPDM. This behavior is correlated with the heterogeneous nature of the blends, which affects the extent of crosslinking and degradation. γ-Irradiation induces chain scission, crosslinking, and oxidation of –CH_2_ groups, reducing their concentration and hence lowering absorbance at 2850 cm^−1^ and 2920 cm^−1^. The interactions between SBS and EPDM from the polymer blends modulate the crosslinking density and the extent of oxidative degradation, resulting in the observed linear decrease in absorbance ratios with increasing doses.

The peaks at 1020 cm^−1^ and 1090 cm^−1^ in the IR spectra are typically associated with stretching vibrations involving C–O stretching, C–C aliphatic stretching, or C–O–C asymmetric stretching bonds [64,65]. In the context of EPDM and SBS polymers, these bands could indicate specific structural features or modifications related to irradiation. For the non-irradiated samples that are rich in aliphatic hydrocarbons, the peaks at 1020 cm^−1^ and 1090 cm^−1^ may correspond to C–C stretching vibrations in the polymer backbone. Possible weak signals from impurities or minor oxidation during sample preparation could be connected with the presence of C–O or C–O–C bonds. When the polymers are exposed to γ-radiation, certain structural changes can lead to the appearance or increase in the intensity of these vibrations due to oxidation, chain scission, and crosslinking. Oxidation and chain scission may create C–O bonds that contribute to the observed peaks, while crosslinking can alter the vibrational modes of the polymer network, potentially enhancing these peaks. In γ-irradiated samples, the presence of new chemical groups and overlapping vibrational modes can result in an additive or intensified effect on the IR peaks. This remark explains why the intensity of the 1020 cm^−1^ and 1090 cm^−1^ bands increases with radiation dose.

The other finding states that the ratio of absorbances A1020/A1090 (Figure 9c) changes on irradiation dose, mostly for the mixtures E25S75, E50S50, and E75S25. It is known that the 1020 cm^−1^ band corresponds to C−O stretching in secondary or tertiary alcohols, while the 1090 cm^−1^ band is linked to C−O stretching vibrations for ether groups or primary alcohols, formed in the polymer matrix due to oxidative degradation of polymer chains [65]. The absorbance ratio A_1020_/A_1090_ ratio increases with dose, indicating that at higher doses, polymers undergo more extensive chain scission and oxidation, forming secondary and tertiary alcohols at a higher rate compared to primary alcohols or ethers. As the radiolysis progresses, the C−O structures associated with secondary/tertiary alcohols dominate, while the contribution of ethers or primary alcohols (1090 cm^−1^) becomes relatively less significant, thus driving the ratio upwards.

Figure 9d shows that the absorbance measured at 1600 cm^−1^ for the tested samples follows a linear trend with SBS content (R^2^ = 0.987). This behavior would be expected because the absorption at 1600 cm^−1^ is primarily due to the stretching of aromatic C=C bonds, which are characteristic of the styrene units in SBS. As the SBS content increases, the number of aromatic rings also increases, leading to higher absorbance [61]. However, at higher irradiation doses (100 and 150 kGy), the absorbance values at 1600 cm^−1^ deviate from the linear trend observed in non-irradiated samples and those irradiated at 50 kGy (Figure 9d). This survey suggests that γ-irradiation progressively alters the structure of the polystyrene aromatic rings. The C=C bonds in the aromatic rings are broken, reducing the absorbance intensity at 1600 cm^−1^ as the aromatic rings are damaged or destroyed. Additionally, irradiation-induced crosslinking between polymer chains or oxidation may further modify the structure of the aromatic rings, either dampening the intensity of their stretching vibrations or forming new chemical structures that absorb differently in this spectral region.

### 3.5. DSC Analysis

The use of chemiluminescence (CL) tests, both isothermal and non-isothermal, combined with DSC data, is considered an effective approach for evaluating degradation behavior. CL, being a highly sensitive technique for detecting oxidative degradation, offers valuable insights into the thermal stability of polymers. By correlating CL data with DSC results, trends in thermal stability can be inferred, particularly when oxidative processes play a major role in degradation.

**Figure 10:**
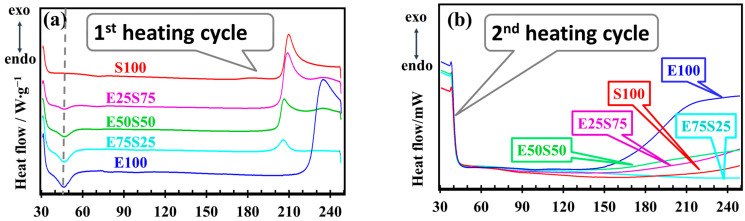
DSC analysis for non-irradiated samples in (**a**) 1st heating cycle, and (**b**) 2nd heating cycle (dark blue line—E100, blue line—E75S25, green line—E50S50, purple line—E25S75, red line—S100).

Figure 10 shows the behavior of the materials when they are heated in the air from 30 to 250 °C. The upper-temperature limit of 250 °C was selected based on the assumption that the polymer will not ever be exposed to temperatures higher than this during processing or use.

In the first heating cycle, an endothermic peak appears at ~40 °C in the compounds containing EPDM. This small peak is absent in SBS and is attributed to the relaxation of polymer chains in the amorphous regions of the polymer. EPDM is more flexible and mainly amorphous, indicating that its polymer chains can undergo local relaxation at lower temperatures, which requires the absorption of heat [66]. As the SBS content increases, the rigid styrene segments from SBS become more dominant in the mixture. These rigid structures limit the chain mobility of the polymer, reducing or eliminating the ability for the chains to relax at ~40 °C. This observation may explain why the endothermic peak decreases and disappears as the SBS content increases, particularly in the S100 sample. SBS itself does not show this relaxation behavior. Instead, it presents a glass transition (Tg) around 75 °C, attributed to the resettlement or rearrangement of the styrene units in the SBS structure. This transition is observed in S100 and E25S75, but not in other compositions.

EPDM shows a large exothermic peak above 220 °C. This peak likely represents an oxidation or crosslinking process, which occurs at high temperatures in EPDM. The composites containing SBS (E75S25, E50S50, E25S75, S100) show this exothermic peak at slightly lower temperatures (above 200 °C). Its size increases with higher SBS content. It suggests that SBS contributes to this process, possibly due to its styrene segments undergoing some form of oxidation or crosslinking at these temperatures. In the second heating cycle, the exothermic peak no longer appears for the samples containing SBS (E75S25, E50S50, E25S75, E100). This assigning indicates that any oxidation or crosslinking processes that could have started in the first heating cycle have already been completed or stabilized. The absence of the exothermic peak in the second cycle indicates that these processes have reached equilibrium or have been suppressed by the presence of SBS. However, in the E100 sample, the exothermic peak reappears in the second heating cycle but is larger and shifted to a lower temperature (~150 °C). The change in the behavior of the investigated compounds from the first cycle (starting at 220 °C) to the second cycle (starting at 150 °C) suggests that EPDM undergoes a more significant reaction (such as crosslinking or oxidation) after the first cycle, which is activated during the second cycle at lower temperatures.

**Figure 11:**
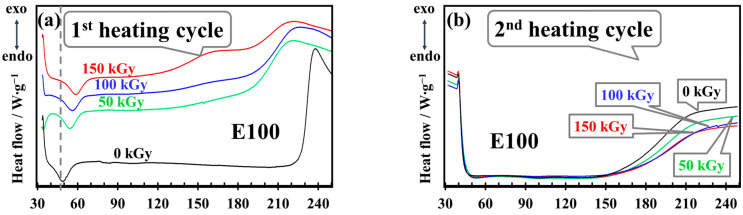

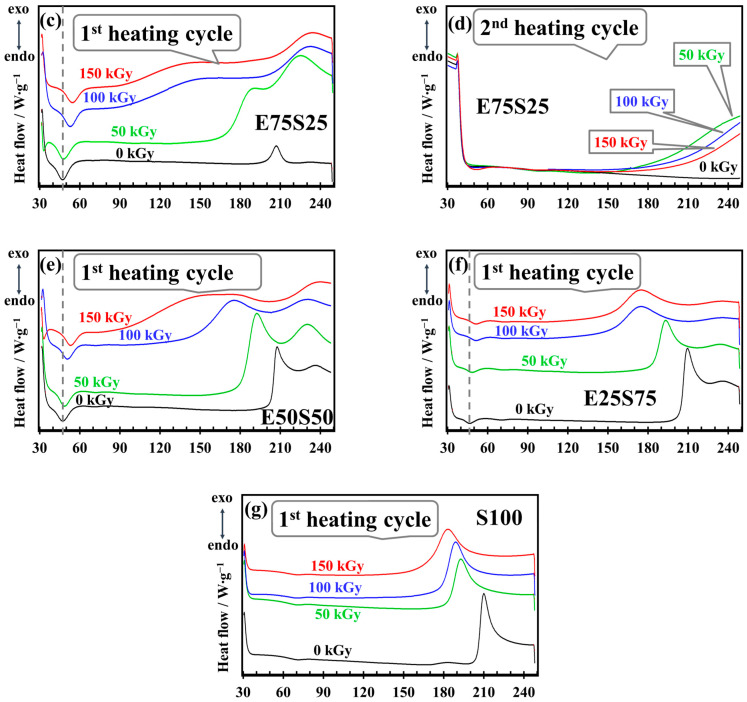
DSC curves for irradiated and non-irradiated samples: (**a**) E100 in the 1st heating cycle, (**b**) E100 in the 2nd heating cycle, (**c**) E75S25 in the 1st heating cycle, (**d**) E75S25 in the 2nd heating cycle, (**e**) E50S50 in the 1st heating cycle, (**f**) E25S75 in the 1st heating cycle, and (**g**) S100 in the 1st heating cycle (black line—0 kGy, green line—50 kGy, blue line—100 kGy, red line—150 kGy).

**Figure 12:**
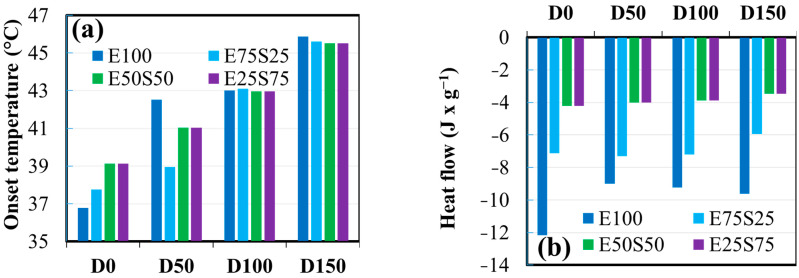

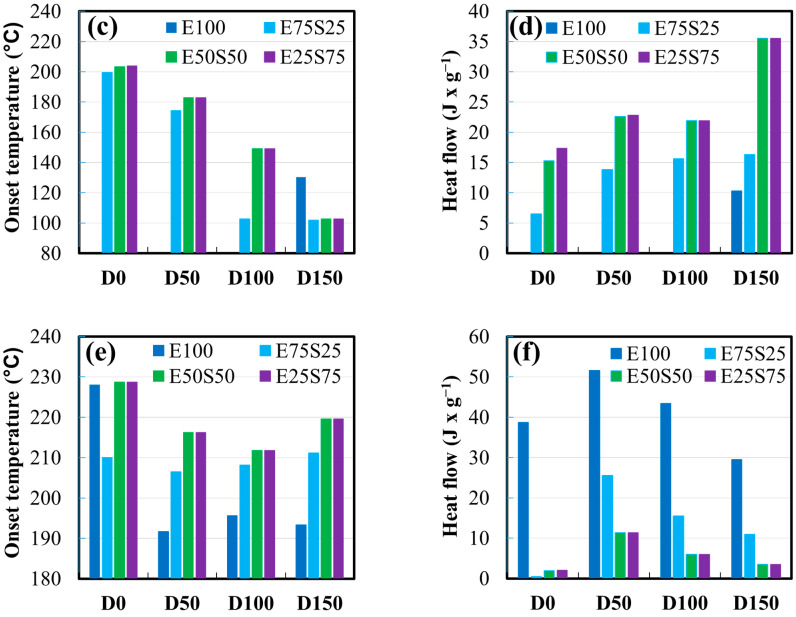
DSC analysis for samples that contain EPDM treated with different irradiation doses, comparing data from the 1st heating cycle: (**a**) onset temperature, and (**b**) normalized heat flow for the endothermal effect; (**c**) onset temperature, and (**d**) normalized heat flow registered for the first exothermal peak; (**e**) onset temperature, and (**f**) normalized heat flow registered for the second exothermal peak.

The observed changes in the samples are reflected in the broadening and shifting of exothermal and endothermal peaks. These findings, derived from the data presented in Figure 11 and Figure 12, are summarized below by sample composition:

**E100**—non-irradiated samples exhibit a sharp exothermal peak above 220 °C (Figure 11a). After irradiation, this peak broadens significantly, indicating a wider range of reaction products caused by structural modifications. At a high irradiation dose (150 kGy), the peak splits into two distinct maxima (humps) at ~130 °C and ~200 °C, suggesting the formation of new reaction products (Figure 12e,f). In the second heating cycle (Figure 11b), thermal effects are almost entirely absent, indicating irreversible structural changes that limit the material’s recyclability or reuse above ~150 °C. These results show that E100 undergoes extensive degradation, crosslinking, and other modifications under irradiation, making it highly susceptible to structural changes.

**E75S25**—non-irradiated samples present a single sharp exothermal peak at ~200 °C (Figure 11c). Following irradiation, a second hump appears at a lower temperature (~150 °C), similar to E100, but at a lower radiation dose (50 kGy). This suggests that adding SBS accelerates structural modifications. The second hump is more prominent than the first, and its normalized heat flow increases with a dose of 50 kGy but decreases at higher doses (100 and 150 kGy), likely due to stabilization or further decomposition of reaction products (Figure 12c–f). In the second heating cycle, only irradiated samples show a broad peak beginning at ~150 °C, indicating irreversible changes in the structure (Figure 11d).

**E50S50** and **E25S75**—these blends exhibit similar behavior, with two exothermal maxima above 150 °C during the first heating cycle (Figure 11e,f). As the SBS content increases, the second exothermal maximum becomes less pronounced, indicating that SBS contributes to stabilization (Figure 12c–f). Increasing the irradiation dose causes both peaks to broaden and shift toward lower temperatures, demonstrating that SBS mitigates but does not completely prevent structural degradation under irradiation.

**S100**—non-irradiated samples display a sharp exothermal peak at ~207 °C (Figure 11g). After irradiation, the peak shifts to lower onset temperatures (170 °C at 150 kGy) but remains sharp, with no additional humps. This indicates that SBS is less prone to crosslinking or significant structural modifications compared to EPDM. The normalized heat flow increases slightly with irradiation, reflecting SBS’s relative stability. Additionally, the absence of thermal effects in the second heating cycle confirms that SBS retains stability even after initial thermal treatment.

Blending SBS with EPDM significantly enhances the thermal and irradiation stability of the material. E100 is highly vulnerable to irradiation-induced changes, which limit its applicability in radiation-exposed environments. In contrast, SBS demonstrates superior stability, making it an essential component for improving the performance of polymer blends. However, the structural changes induced by irradiation, particularly in EPDM-rich compositions, constrain the reusability of these materials, especially at higher doses.

## 4. Conclusions

This study investigates the effect of γ-irradiation on elastomer blends of SBS and EPDM in various weight ratios to determine an optimal blend for applications in oncology devices, nuclear power plants, and space exploration where materials face unprotected exposure to radiation. A combination of chemiluminescence testing, sol-gel analysis, mechanical testing, IR spectra, and DSC analysis was employed to evaluate the material properties and irradiation effects.

The results showcase a trade-off between the stability provided by SBS and the flexibility offered by EPDM. Combining EPDM and SBS in a polymer blend enhances the properties of the material. EPDM imparts excellent weather resistance and flexibility, while SBS contributes to toughness and ease of processing. Sol-gel experiments revealed that the non-irradiated samples dissolved fully in toluene, while irradiated samples (50, 100, and 150 kGy) produced a gel fraction in blends with higher EPDM content (E100, E75S25, E50S50). In contrast, samples with higher concentrations of SBS (E25S75 and S100) dissolved completely, regardless of irradiation dose.

FTIR, CL, and DSC analyses demonstrated that SBS concentrations exceeding 50% in EPDM/SBS blends result in materials with exceptional resistance to high radiation doses. Interestingly, as the irradiation dose increased (100 and 150 kGy), the absorbance at 1600 cm^−1^ deviated from the trend observed at lower doses, suggesting structural changes in the polystyrene aromatic rings as a result of γ-irradiation.

The blend with 75% SBS (E25S75) exhibited the best performance under γ-irradiation, showing superior resistance to radiation-induced mechanical and structural degradation. This makes E25S75 a strong candidate for high-radiation environments, thanks to its physical and mechanical stability. The findings confirm that γ-irradiation induces controlled degradation in SBS/EPDM mixtures: EPDM suffers higher crosslinking, whereas SBS resists crosslinking, thus improving the stability of EPDM under irradiation.

Thermal analysis further highlights the stabilizing effect of SBS in the blends. SBS-rich samples (E25S75, E50S50, S100) demonstrated enhanced thermal stability under γ-irradiation, characterized by reduced broadening of exothermal peaks, lower onset temperatures, and minimal shifts in thermal behavior during consequent heating cycles. The inclusion of SBS mitigates irradiation-induced degradation by reducing crosslinking and chain scission, resulting in enhanced consistency in thermal properties across cycles. Overall, SBS emerges as an effective stabilizing component in EPDM/SBS blends, particularly in high-radiation conditions. The blend with 75% SBS shows optimal performance, combining high thermal and radiation stability with mechanical durability.

## Figures and Tables

**Table 1:** The activation energies for the oxidation of unirradiated EPDM/SBS blends.

Material	OIT (min)			Correlation Factor	Activation Energy (kJ mol^−1^)
	160 °C	170 °C	180 °C		
E 100	98	30	11	0.99940	90
E75S25	70	35	10	0.98428	80
E50S50	44	18	7	0.99958	76
E25S75	48	25	9	0.99025	69
S 100	38	16	9	0.94750	60

**Table 2:** The values of onset oxidation temperatures for γ-irradiated EPDM/SBS blends.

Ageing Procedure (Radiation Dose)	OOT (°C)				
	E100	E75S25	E50S50	E25S75	S100
0 kGy	171	206	211	222	220
50 kGy	162	182	192	212	215
100 kGy	144	165	163	205	201
150 kGy	109	112	135	184	183

**Table 3:** The results of the swelling investigation.

Sample	Dose (kGy)	Gel Fraction (%)	*V_r_*	ρ_r_ (g/cm^3^)	ν_cross_ × 10^−4^ (mol cm^−3^)	*p/q*
E_50_S_50_	50	41.81 ± 0.003	0.1166 ± 0.003	0.9219 ± 0.002	0.0291 ± 0.005	0.1927
	100	70.17 ± 0.067	0.1713 ± 0.006	0.9427 ± 0.004	0.1940 ± 0.027	
	150	85.24 ± 0.013	0.2336 ± 0.013	0.9450 ± 0.003	0.6543 ± 0.133	
E_75_S_25_	50	75.12 ± 0.016	0.1364 ± 0.004	0.8699 ± 0.002	0.1412 ± 0.015	0.1468
	100	93.37 ± 0.007	0.2966 ± 0.002	0.8932 ± 0.011	1.8319 ± 0.238	
	150	98.50 ± 0.001	0.3366 ± 0.002	0.9116 ± 0.002	2.7576 ± 0.064	
E_100_	50	98.53 ± 0.001	0.2728 ± 0.006	0.8878 ± 0.002	1.6316 ± 0.105	0.084
	100	99.27 ± 0.001	0.3647 ± 0.002	0.8898 ± 0.004	4.0706 ± 0.073	
	150	98.98 ± 0.001	0.3879 ± 0.001	0.8950 ± 0.001	4.9850 ± 0.023	

## Data Availability

The original contributions presented in this study are included in the article/Supplementary Material. Further inquiries can be directed to the corresponding authors. Correspondence and requests for materials should be addressed to T.Z. and M.B.

## Supplementary Materials

The following supporting information can be downloaded at: https://www.mdpi.com/article/10.3390/polym17101314/s1, Table S1. Formulation of the blends.

## Abbreviations

The following abbreviations are used in this manuscript:

CL	Chemiluminescence
DSC	Differential scanning calorimetry
EPDM	ethylene-propylene-diene monomer
FTIR	Fourier transformed infrared
PP	polypropylene
SBS	styrene-butadiene-styrene
