# Supplementary material for: Evaluation of γ-Irradiation Effects on EPDM/SBS Blends for Durability and Recycling Potential
Source: Polymers (Basel). 2025 May 12;17(10):1314. doi: 10.3390/polym17101314 (PMC12114983; doi:10.3390/polym17101314)
Supplement: Supplementary file 1 [file polymers-17-01314-s001.zip › polymers-3588983-supplementary.pdf]

polymers

MDPI

Article – Supplementary material

Supplementary material

Evaluation of γ-Irradiation Effects on EPDM/SBS Blends for Durability and Recycling Potential

Traian Zaharescu [1,2], Marius Bumbac [3,*], Cristina Mihaela Nicolescu [4,*], Maria Daniela Stelescu [5], Tunde Borbath [2] and Istvan Borbath [2]

1 Radiochemistry Center, National Institute for Research and Development in Electrical Engineering ICPE-CA, RO 030138 Bucharest, Romania; traian.zaharescu@icpe-ca.ro
2 SC Roseal SA, RO 535600 Odorheiu Secuiesc, Romania; borbath.tunde@gmail.com (T.B.); borbathistvan@roseal.eu (I.B.)
3 Faculty of Science and Arts, Valahia University of Targoviste, RO 130004 Targoviste, Romania
4 Institute of Multidisciplinary Research for Science and Technology, Valahia University of Targoviste, RO 130004 Targoviste, Romania
5 Leather and Footwear Division, National Research and Development Institute for Textiles and Leather, RO 031215 Bucharest, Romania; dmstelescu@yahoo.com
* Correspondence: marius.bumbac@valahia.ro (M.B.); cristina.nicolescu@valahia.ro (C.M.N.); Tel.: +40-721219270 (M.B.); +40-722246416 (C.M.N.)

S1. The Composition and Codification of Analyzed Samples

The samples were prepared by the melt blending technique using an electrically heated laboratory roller mill machine, model ZG-160YRDB (from Xiamen Ollital Technology Co Ltd, Xiamen, China). The working parameters were: friction ratio 1:1.2, temperature 95-115°C, and blending time 10 minutes for each formulation.

The sample compositions are listed in Table 1.

Table S1. Formulation of the blends.

Components	Blending percentage (wt%)				
	S100	E25S75	E50S50	E75S25	E100
EPDM	-	25	50	75	100
SBS	100	75	50	25	-
TPTMA	3	3	3	3	3

S2. Chemiluminescence Study—Activation Energy Calculation

The activation energies required for the oxidation of the studied samples were calculated by the Arrhenius procedure [1] using equation (1).

$$\text{tg}\,\alpha = \text{-E\_a/R} \qquad \text{(S1)}$$

where tg α is the slope of ln   vs reciprocal temperature, Ea is the value of activation energy (kJ mol^{-1}), R is the ideal gas constant (8.314 J $K^{-1}mol^{-1}$).

S3. Volume Fraction of Rubber

V_r is the volume fraction of rubber in swollen mass calculated with the equation (2).

$$V_r = \frac{\text{volume of rubber}}{(\text{volume of rubber})+(\text{volume of solvent})} = \frac{\frac{m_d}{\rho_r}}{\frac{m_d}{\rho_r}+\frac{m_s-m_d}{\rho_s}} \quad \text{(S2)}$$

where: ρ_s - density solvent (0.866 g/cm^3 for toluene), ρ_r - densities of the dry rubber sample, and m_s-m_d = weight of solvent in the swollen rubber [2]. The density of elastomer samples was measured according to ISO 2781.

For our evaluation of crosslinking densities we ascribe the values of 0.496 for the interaction parameter EPDM/toluene [3] and 0.570 for the interaction parameter SBS/toluene [2]. For the blends the weighted average of the polymer/solvent interaction parameter (χ_{12}): for E25S75: 0,551, for S50E50: 0,533 and for E75S25: 0.514.

References

1. Zaharescu, T. Synergistic effect of silica nanoparticles assisted by rosemary powder in the stabilization of styrene-isoprene-styrene triblock copolymer. *Radiat. Phys. Chem.* **2023**, *206*, 110765.
2. Díez, E.; Ovejero, G.; Romero, M.D.; Díaz, I. Polymer-solvent interaction parameters of SBS rubbers by inverse gas chromatography measurements. *Fluid Phase Equilibria* **2011**, *308*, 107–113.
3. Pirityi, D.Z.; Pölöskei, K. Thermomechanical devulcanisation of ethylene propylene diene monomer (EPDM) rubber and its subsequent reintegration into virgin rubber. *Polymers* **2021**, *13*, 1116.

Disclaimer/Publisher's Note: The statements, opinions and data contained in all publications are solely those of the individual author(s) and contributor(s) and not of MDPI and/or the editor(s). MDPI and/or the editor(s) disclaim responsibility for any injury to people or property resulting from any ideas, methods, instructions or products referred to in the content.
